# Composite Hyaluronic Acid Gas-Entrapping Materials to Promote Wound Healing

**DOI:** 10.1021/acs.biomac.4c00904

**Published:** 2025-01-02

**Authors:** Emily Witt, Emily B. Petersen, Eyas Alzayadneh, Ryan J. Courtney, Marc J. Brouillette, Qi Wang, Maxwell Y. Sakyi, Nicole A. D. Watson, Dominic Rivas, Jianling Bi, Lindsey Culver, Kyle Balk, Colin Reis, Slyn Uaroon, Kaitlyn A. McClintic, Samual Hatfield, Kristan S. Worthington, Edward A. Sander, Giovanni Traverso, Leo E. Otterbein, Jessica E. Goetz, Douglas C. Fredericks, James D. Byrne

**Affiliations:** † Department of Biomedical Engineering, University of Iowa, Iowa City, Iowa 52242, United States; ‡ Department of Radiation Oncology, University of Iowa, Iowa City, Iowa 52242, United States; § Department of Pathology, University of Iowa, Iowa City, Iowa 52242, United States; ∥ Department of Orthopedics and Rehabilitation, University of Iowa, Iowa City, Iowa 52242, United States; ⊥ Carver College of Medicine, University of Iowa, Iowa City, Iowa 52242, United States; # Department of Otolaryngology, University of Iowa, Iowa City, Iowa 52242, United States; ∇ Division of Gastroenterology, Brigham and Women’s Hospital, Harvard Medical School, Boston, Massachusetts 02115, United States; ○ Department of Mechanical Engineering, Massachusetts Institute of Technology, Cambridge, Massachusetts 02139, United States; ◆ Department of Surgery, Beth Israel Deaconess Medical Center, Harvard Medical School, Boston, Massachusetts 02215, United States; □ College of Korean Medicine, Kyung Hee University, Seoul, 02447, Republic of Korea

## Abstract

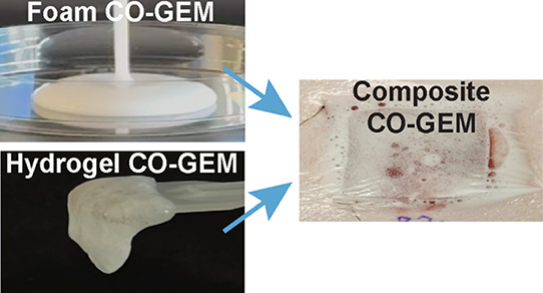

Tissue repair is often impaired in pathological states, highlighting the need for innovative wound-healing technologies. This study introduces composite hyaluronic acid gas-entrapping materials (GEMs) delivering carbon monoxide (CO) to promote wound healing in pigs. These composite materials facilitate burst release followed by sustained release of CO over 48 h. In a porcine full-thickness wound model, CO-GEMs significantly accelerated wound closure compared to the standard-of-care dressing (Tegaderm). Wound area closure with CO-GEMs was 68.6% vs 56.8% on day 14, 41.0% vs 25.1% on day 28, and 26.9% vs 11.8% on day 42, effectively reducing healing time by 14 days. Histological analysis revealed increased epithelialization and neovascularization with reduced inflammation. These findings demonstrate the potential of CO-GEMs as a topical therapeutic to enhance tissue repair in clinically relevant models, supporting further testing for wound-healing applications.

## Introduction

The skin, composed of the epidermis, dermis, and subcutaneous layers, is the body’s largest organ. It serves as a protective barrier, aids in homeostasis, and functions as a sensory interface.^1^ Damage to the skin can lead to infection and significant health risks, especially for vulnerable populations.^2^ The process of wound healing varies depending on wound size, depth, prior debridement, nutritional or immunological deficiencies, age, chronic stress, and other comorbidities.^3^ As the global population ages, the incidence of chronic diseases and chronic wounds is increasing, creating a substantial economic burden. Treating chronic wounds, such as pressure and venous ulcers, as well as surgical and traumatic injury to the skin remains a significant health challenge, affecting between 2.4 and 4.5 million people and costing $20 billion annually in the United States.^4^ Treatments including wound dressings, cell transplantations, and negative pressure therapies have shown inconsistent outcomes and present logistical barriers due to consistent access to these therapies.^1,5−7^

Recent studies have highlighted the potential of topical gas therapies in modulating the wound healing process.^8−13^ Previous studies, including our own, have demonstrated that topical gas therapies foster local vasodilation, angiogenesis, and reduce oxidative stress, synergistically promoting wound healing and reducing bacterial growth.^12−14^ Specifically, there is interest in using carbon monoxide (CO), a gasotransmitter known for its deleterious, as well as anti-inflammatory, antimicrobial, immunomodulatory, and pro-vascular effects, to accelerate wound healing.^15^ We have previously generated simple hyaluronic acid-gas-entrapping materials (GEMs) that rapidly release CO and found that daily topical administration on wounds resulted in significant acceleration of wound healing compared to nitrogen-releasing GEMs or no treatment in diabetic wound models in mice.^13^

To prolong exposure of CO and reduce the frequency of reapplication of GEM, we created a novel composite system that facilitates an initial burst release followed by a prolonged release of CO. We hypothesized that CO-GEMs would improve healing in a more clinically relevant model and thus tested the wound healing capacity of these systems in a porcine full-thickness wound model. Similar to that observed in mice, we observed a dramatic acceleration of wound healing with the application of CO-GEMs compared to Tegaderm Transparent Film, a 3M standard-of-care dressing.

## Materials and Methods

### Study Design

This study aimed to assess the effectiveness of prolonged topical carbon monoxide (CO) delivery for wound healing. Generally Recognized As Safe (GRAS) components were assembled into gas-entrapping materials to deliver CO. These GEMs were characterized *in vitro* and tested for their ability to modulate and improve wound healing *in vivo* in a porcine full-thickness wound model. In addition, we measured wound closure rates, histological markers of healing, and inflammatory responses. Animal protocols were approved by the Institutional Animal Care and Use Committee at the University of Iowa (4032581). The researcher responsible for wound measurements was blinded to the treatment groups, and the pathologist who performed the histological analysis was blinded to the study groups. All animals were included in the analysis.

### Fabrication of CO-GEMs

The foam CO-GEM was prepared according to a previously described method.^13,16 −18^ In brief, a prefoam solution was prepared by adding 0.8 wt %age (wt %) methylcellulose (Modernist Pantry) and 1.25 wt % high molecular weight hyaluronic acid (Bulk Naturals) to 400 mL (mL) 1X PBS (phosphate buffered saline). The solution was heated to 100 °C while stirring until completely dissolved. Once cooled, the prefoam solution was subjected to autoclaving and degassing for over 8 h before use. One hundred mL of prefoam solution was added to a modified iSi 1-pint whipping siphon equipped with a custom-made M22–1/4 NPT connector for attachment to gas regulators. The whipping siphon was pressurized to 200 pounds per square inch (PSI) with 100% CO (Linde) and then shaken for 30 s before use.

The hydrogel GEMs were prepared using a thiolated dithiopropionylhydrazide (DTPH)-modified hyaluronic acid and poly(ethylene glycol) diacrylate (PEGDA) available through Advanced Biomatrix (Cat no. GS1004F). The two-part hydrogel system is commonly used for *in vitro* stem cell work and was modified to create CO-GEMs.^19^ Both components were reconstituted after reaching room temperature and combined in a 4:1 ratio of thiolated dithiopropionylhydrazide (DTPH)-modified hyaluronic acid):(poly(ethylene glycol) diacrylate (PEGDA). The solution was intermittently mixed every 5 min. At 17 min after the initial combination, 5 mL of the viscous solution was loaded into a 10 mL syringe, the syringe connected to a syringe adapter and mixed at least 10 times with a separate syringe filled with 5 mL CO gas (Linde) until the gas was fully incorporated. The gasified hydrogel was then inserted into a 5 × 5 cm silicone tray, and the tray was placed in a gastight sample storage container (Rave Scientific) and purged with 5 PSI CO for 5 min. After 5 min, the container was sealed, and the hydrogel GEMs were allowed to solidify fully for 2 h at room temperature before freezing.

### Characterization of CO-GEMs

The GEMs were analyzed and characterized at both macroscopic and microscopic levels. An EVOS microscope at 10× magnification was used to image CO-GEMs. To quantify CO entrapment and CO release from the foam CO-GEMs, 5 mL of the foam was placed into borosilicate glass gas-chromatography (GC) vials and then sampled at 0.5, 1, 3, 6, 24, and 48 h. To quantify CO entrapment and CO release from the hydrogel CO-GEMs, 5 mL of the hydrogel CO-GEMs was placed into a GC vial and allowed to solidify for 2 h before freezing; the GC vials were subsequently exposed to ambient conditions during thawing process to ensure accurate gas release and measurement from the hydrogel. The GC vials containing the hydrogels were then capped and then sampled at 0.5, 1, 3, 6, 24, and 48 h. All samples underwent analysis using the GC-thermal conductivity detector, with calibration curves generated using the same 99.3% CO cylinder used to create the CO-GEM.

Hydrogel samples were cast into a dog bone shape using a 3D-printed casting tray mimicking the shape of the skin samples, resulting in specimens that were 4 mm wide, 8 mm long, and 5 mm thick in the gauge region. After casting, the hydrogels were briefly frozen, thawed, and then extracted from the casting tray. The specimens were placed into custom 3D-printed tissue clamps to interface with the electromechanical material testing machine (MTS Insight, MN, USA). Unlike the skin samples, no preload was applied to these specimens due to their delicate nature. Instead, the clamp separation distance was maintained when installing the specimen into the MTS using a guide fixture. A load-to-failure test was performed using the MTS system. The specimen clamps were rigidly secured, and the hydrogels were uniaxially stretched at a strain rate of 0.01 s^–1^, with time, displacement, and load data recorded at 100 Hz for subsequent analysis. The analysis of the hydrogels. Linear stiffness (Newtons (N)/mm), maximum load at failure (N), maximum extension or displacement at failure (mm), and the energy absorbed by the hydrogels at failure (N-mm) were calculated. Due to the lower signal-to-noise ratio in the hydrogel data, linear stiffness was derived from a line fit to a 2 mm section of displacement data before any failure event. Maximum load was the highest force reached, which defined the failure point and the corresponding maximum extension. Energy absorbed was calculated as the area under the displacement versus load curve up to the point of maximum extension.

Rheology was performed using Kinexus Ultra+ (Malvern Panalytical, Malvern UK). For each formulation, a frequency sweep from 0.1 to 20 Hz (0.65 rad/s to 130 rad/s; 1% strain controlled) was performed at 37 °C for 3 replicates with roughened stainless steel 20 mm diameter upper and lower parallel plates. Foam gels were lightly loaded (<0.05 N) to a 1.5 mm gap height. Frozen hydrogels were thawed for 1 h at room temperature and measured by lowering the upper geometry until a nominally small force (∼0.06 N) was observed, with gap heights ranging from 1.55 to 1.7 mm. Foam gels and frozen hydrogels were trimmed to the edge of the upper plate geometry before each run. Storage modulus, loss modulus, and tan-delta were exported from Malvern Panalytical software.

### *In Vitro* Studies

Human dermal fibroblast cells, sourced from a 28-year-old male via the Coriell Institute, were seeded onto 96-well plates at a density of 6,000 cells per well. After 24 h, media was mixed with a hyaluronic acid–based prefoam solution. Cell viability at 72 h was measured using the alamarBlue assay (Thermo Scientific), following the manufacturer’s instructions. Absorbance was recorded using a microplate reader (Bio-Rad Laboratories) with a 560/590 nm (excitation/emission) filter setting. The data were normalized against the untreated control, set at 100% viability.

For the scratch assay, human dermal fibroblasts were plated in a 24 well plate at 50,000 cells/well within inserts from an Abcam Wound Healing Assay Kit to create wounds. Following a 12-h incubation period, the insets were carefully removed with sterile tweezers to expose the wound site. Control plates were placed in a conventional incubator, while the experimental plates were placed in hypoxia chambers with 250 ppm of CO. Images were captured at 18 h postincubation to assess wound healing progression, using a 4*x*/0.16 magnification objective. Wound areas were quantified using a scale bar and analyzed with ImageJ software.

### Evaluation of CO-GEMs in a Porcine Full-Thickness Wound Model

Male Yorkshire/Landrace hybrid pigs (Premiere BioSource) aged 16 weeks were acclimated to the facility prior to the procedure. To create the full-thickness model, the pigs were anesthetized, and hair was clipped from the surgical area. The skin was subsequently cleaned with alternating 2% chlorhexidine scrub and 70% isopropyl alcohol followed by a final sterile prep of 2% chlorhexidine gluconate and 70% isopropyl alcohol solution. To create the wound, a 5 × 5 cm square sterile stencil was traced onto the dorsum of the pig using a sterile tissue marker. Sterile disposable #21 scalpels were used to excise the full dermis down to the fascia, and the 5 × 5 cm skin piece was removed. A single stitch with 4–0 silk suture was placed in the intact skin at each corner of the wound to represent the initial boundaries of the wound. A total of 8 wounds were created per pig, 4 per side, separated by 4 cm on the cranial, medial, and caudal sides. On the day of wound creation and each day of treatment, the skin surrounding the wounds was cleaned with sterile saline and gauze. A picture was taken of each wound with a measurement tool for accurate size recording. Prior to the application of the CO-GEMs, Cavilon No Sting Barrier Film was applied to the skin surrounding each wound. On days 0, 2, 4, 6, 8, and 10, a volume of 8 mL of foam CO-GEM was applied directly to the wound. This was then covered with a 5 × 5 x 0.5 cm (length x width x height) hydrogel CO-GEM. Each wound was bandaged individually with Tegaderm to maintain the GEM position over the wound. On days 14, 16, 18, 21, 25, 28, 32, 35, and 38, a volume of 8 mL of foam CO-GEM was applied directly to the wound. The foam and wound area were bandaged individually with Tegaderm to maintain GEM positioning over the wound area. The control wounds were cleaned, photographed, and covered with Tegaderm in the same fashion as the CO-GEM-treated wounds, except no additional wound healing agent was applied onto the wound. Four 12 × 12 cm gauze pads were applied over the wounds, and stretch netting was applied to keep the gauze pads in place. A Spandex sheep tube was applied over the netting to keep the bandages in place. Pigs were euthanized on Day 42 following a final wound evaluation.

For wound measurement, ImageJ (v. 1.54d, Java 1.8.0_345) was used to analyze the wound images. The perimeter of each wound area was traced using the polygon tool and the area was measured to scale. During each treatment and bandage exchange, 1 mL of whole blood was collected in 1 mL BD syringes (prefilled with 100 units of heparin) prior to each application of CO-GEMs or prior to bandage removal in the control group, 15 min after CO-GEM application or at the end of bandage exchange in the control group, and 1 h after CO-GEM application or after bandage exchange. Blood was analyzed using a Radiometer ABL80 FLEX CO-OX blood gas analyzer.

For mechanical testing of the porcine skin, full-thickness healed defect samples were stored in saline-soaked gauze at −20 °C until the day of mechanical testing. On the testing day, the skin samples were fully thawed, and cutting guides were used to excise dumbbell-shaped specimens that were 4 × 8 mm in the gauge region, which consisted only of newly healed tissue in the samples with surgical defects. Custom 3D-printed clamps and spacing guides were used to ensure consistent clamping distance and fixturing to the mechanical testing machine. An electromechanical material testing machine (MTS Insight, MN, USA) was used to perform a load-to-failure test. Specimen clamps were rigidly secured to the testing machine, and a 0.1 N preload was applied to lightly tension the sample. Ten preconditioning cycles of 0.4 mm displacement (5% strain) at 0.25 Hz were then applied to the tissue, immediately followed by a load-to-failure test at a strain rate of 0.01 s^–1^. Data was recorded at 100 Hz and imported into Matlab R2023 (MathWorks Inc., MA, USA) for analysis using custom code to calculate the structural properties of the tissue: linear stiffness (N/mm), maximum load at failure (N), maximum extension or displacement at failure (mm), and the energy absorbed by the tissue at failure (N-mm), similar to the method described previously.^20^

Linear stiffness was defined as the slope of a line fit to a 1 mm section of the force–displacement curve before any tissue failure event. These data were extracted from the full curve by first calculating the instantaneous slope between adjacent data points and then finding the highest mean slope over a sliding 1 mm window of the instantaneous slope values before a failure event (sudden drop in force), whether partial or full-thickness tissue tear. The maximum load was the highest force reached and defined the failure point at which the maximum extension was reported. The area under the force–displacement curve up to the maximum extension length was defined as the energy absorbed.

## Results and Discussion

### CO-GEMs Are Designed and Engineered to Facilitate and Enhance the Wound Healing Process

CO is a unique gasotransmitter with pleiotropic effects, including immunomodulation and provascularization.^21^ Other gases have been used for wound healing, most notably nitric oxide.^15^ There are unique challenges to using nitric oxide for wound healing, including that it is extremely unstable as a gas, where it is converted to harmful byproducts, which limits its delivery to a carrier molecule or nitrite.^22^ Further, tunable dosing of the gas is limited due to its lability in oxygen-rich/aqueous environments.^11^ Given these challenges and the potential for the positive pleiotropic effects of CO, we were interested in evaluating the impact of a CO-filled wound dressing in a human-relevant model for wound healing. To assess the wound healing capacity of CO in an *in vitro* model, we performed a scratch assay using human dermal fibroblasts. Cells were exposed to 250 ppm of CO or room air and were found to have a significant increase in cell migration at 18 h after wound creation (Figure S1A,B). The exact threshold CO doses for wound healing *in vitro* and *in vivo* are the subject of future studies.

**Figure 1:**
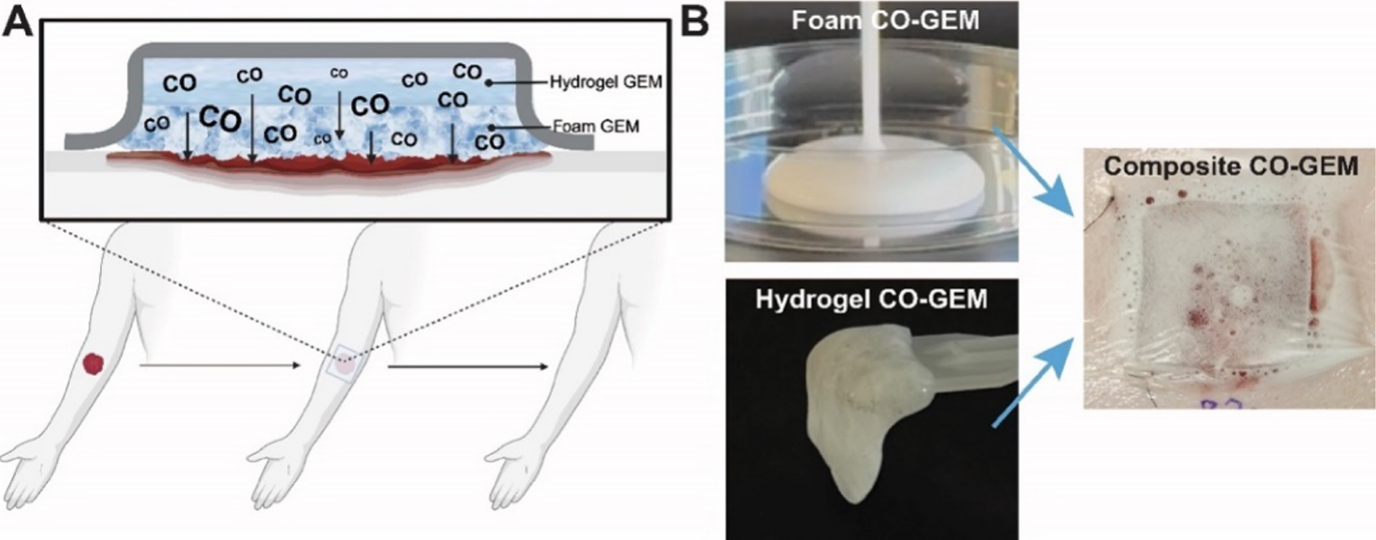
Carbon monoxide gas-entrapping materials (CO-GEM) were engineered for prolonged gas release to promote wound healing. (A) Schematic for the treatment of wounds using topical application of a composite foam and hydrogel GEM. Created in BioRender. Byrne, J. (2024) https://BioRender.com/r84h334. (B) Foam and hydrogel GEMs were combined to create a composite GEM placed on a full thickness porcine wound and covered with Tegaderm.

For ease of administration and reapplication of a CO-filled wound dressing in pigs, we designed and developed composite hyaluronic acid–based GEMs capable of prolonged CO release. Figure 1A,B illustrates the application and composition of the GEMs used for wound healing. Composite CO-GEMs were engineered to facilitate CO release immediately upon application followed by sustained release of CO between reapplications every 48–72 h. This ensured continuous exogenous CO exposure within the wound between each reapplication. High molecular weight hyaluronic acid was chosen as the base material for the CO-GEMs since high molecular weight hyaluronic acid has a demonstrated benefit in wound healing.^23,24^ To validate the lack of toxicity from the base materials, we evaluated cell viability of human dermal fibroblasts with increasing concentrations of prefoam solution and found no cytotoxicity up to 10 mg/mL (Figure S2). The hydrogel was not assessed for toxicity, as it is primarily used for culturing stem cells and has been found to be nontoxic and biocompatible.^19,25^

Next, we generated and tested the material properties of each component of the composite CO-GEMs. The foam CO-GEMs were produced using commercially available whipping siphons (Figure S3A) to physically entrap CO within a foam. To generate hyaluronic acid hydrogels capable of gas release, we gasified thiolated DTPH-modified hyaluronic acid and PEGDA with CO using a syringe-mixing method (Figure S3B). The thiol groups on the thiolated DTPH-modified hyaluronic acid reacted with the acrylate groups on PEGDA to form a covalently cross-linked hydrogel through a thioether bond resulting in a matrix encapsulating the CO. The precured hydrogel was mixed at a 1:1 ratio of the hydrogel:CO to maintain the physical integrity of the hydrogel. The foam and hydrogel CO-GEMs were noted to have different gas bubble distributions (Figure S4A,B), likely as a function of the difference in fabrication process. The foam CO-GEM exhibited self-healing behavior via rheologic assessment, which will allow for ease of administration on the wound (Figure S4C). The hydrogel CO-GEMs were soft with substantial plastic deformation, as found by rheological assessment and mechanical testing (Figure S4D–F). The schematic in Figure 1A demonstrates the application of CO-GEMs to wounds. Figure 1B shows the composite CO-GEM covering the wound. Using hyaluronic acid as a base composition, we have generated soft composite CO-GEMs capable of placement in a wound bed.

### Foam and Hydrogel CO-GEMs Encapsulate Large Amounts of CO, Allowing Sustained Release

**Figure 2:**
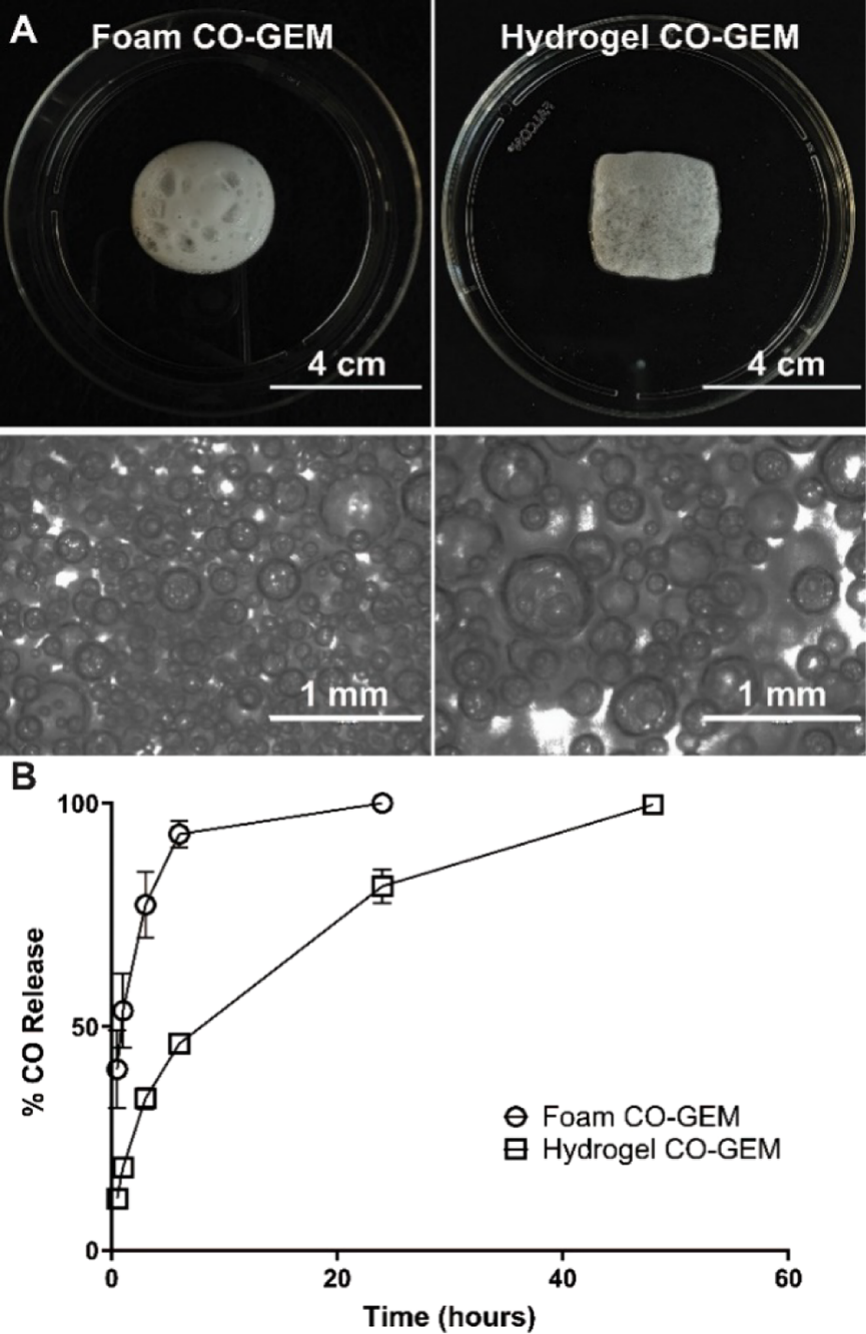
Foam and hydrogel CO-GEM formulations have distinct CO release kinetics. (A) Macroscopic and microscopic images of CO-GEMs. (B) CO release kinetics from each GEM as analyzed by gas chromatography (*n* = 3/time point).

Foam and hydrogel CO-GEMs physically entrap large amounts of CO using non-cross-linked and cross-linked high molecular-weight hyaluronic acid (Figure 2A). We evaluated the concentration of CO in both formulations and found the CO concentration in the foam CO-GEM to be around 1 mg/g, which was approximately 2-fold higher than hydrogel CO-GEMs (0.5 mg/g) (Figure S5). We then tested the CO release kinetics from both formulations. The foam CO-GEMs facilitated a rapid release of CO, with the majority of CO being released in 6 h. In contrast, the hydrogel CO-GEM showed prolonged release of CO over 48 h (Figure 2B). Furthermore, the foam CO-GEMs remain stable at 4 °C prior to dispensing as foams (Figure S6A), and the hydrogel CO-GEMs demonstrated stability at −50 °C prior to thawing (Figure S6B) and stable thickness over the course of 3 days at 37 °C (Figure S6C). In summary, we have created stable CO-GEMs capable of prolonged CO release.

### Topical Administration of Hyaluronic Acid CO-GEMs Significantly Enhances the Rate of Wound Healing in Porcine Models

**Figure 3:**
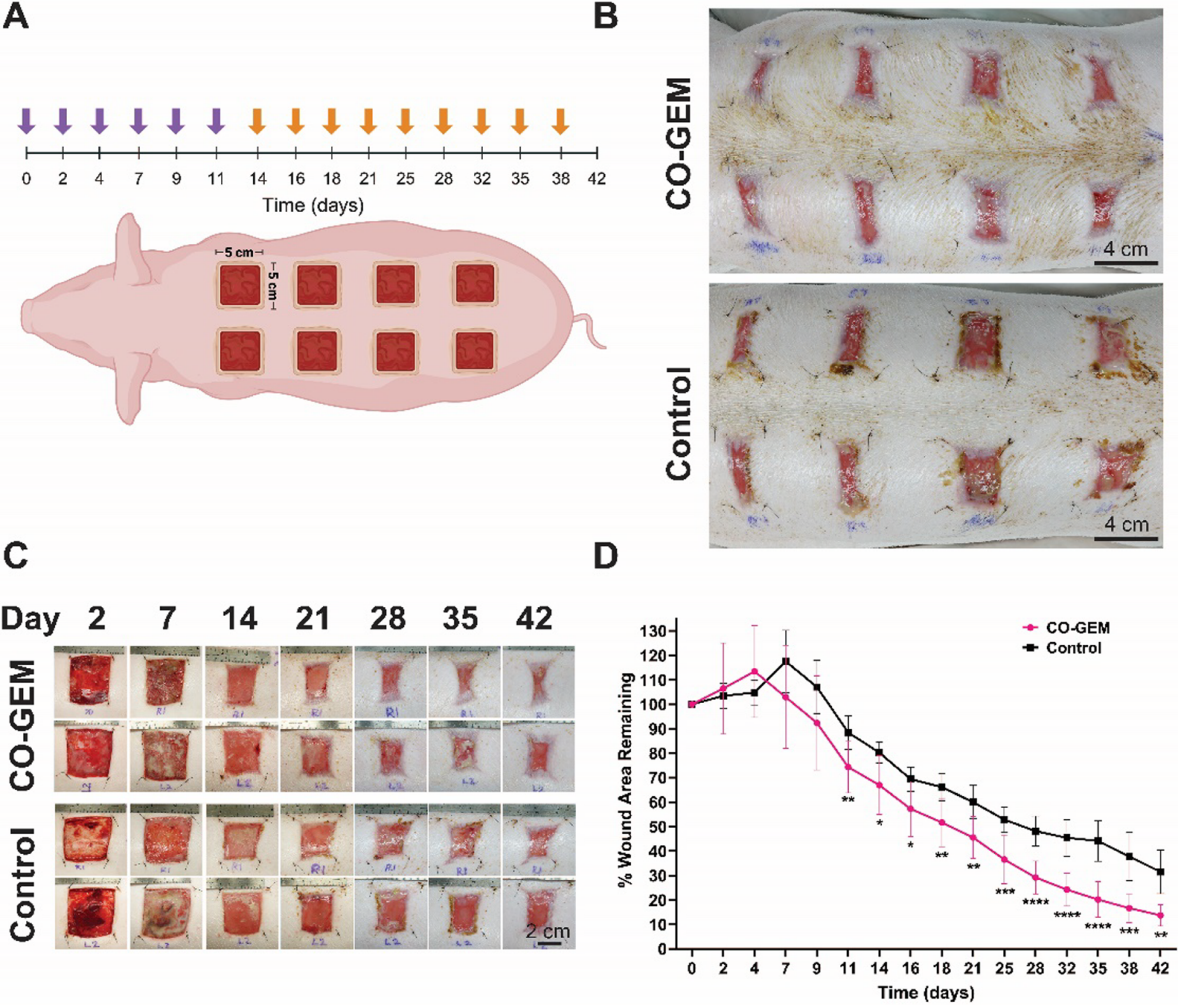
CO-GEMs promote wound healing in full thickness wounds on the dorsum of pigs. (A) Treatment outline for the study where purple arrows indicate treatment with composite CO-GEMs (foams and hydrogels) and yellow-orange arrows indicate treatment with foam CO-GEMs. The treatments were transitioned from composite CO-GEMs to foam CO-GEMs due to residual hyaluronic acid hydrogel remaining and integrating into the wound. Made with BioRender.com. (B) Images of all eight wounds on control pig and pig treated with CO-GEMs at day 38. There is a visible reduction in wound size and new skin over the CO-GEM-treated animals. (C) Single images of wounds from the same position for control and CO-GEM-treated animals. (D) Quantification of wound area remaining using ImageJ for wounds on control pig and CO-GEM-treated pig (*n* = 8 wounds/arm). *p* values were quantified by unpaired *t* test at the specified time point. *p < 0.05, **p < 0.01, ***p < 0.001, ****p < 0.0001.

We next tested the topical application of hyaluronic acid-containing CO-GEMs on full-thickness wounds in pigs. Eight 5 × 5 cm full-thickness wounds were created on the back of two anesthetized Yorkshire/Landrace pigs (Figure 3A). The wounds were immediately treated with composite CO-GEMs and covered with Tegaderm, wound dressings, and netting. The pigs were redosed according to the schedule shown in Figure 3A. Prior to redosing (CO-GEMs) or redressing (Tegaderm), the areas around the wounds were cleaned, and no secondary tissue damage was noted when redosing the wounds. There was a significant improvement in healing observed in CO-GEM-treated wounds compared to control Tegaderm-treated wounds (Figure 3B–D). Control wounds were covered with Tegaderm to keep them moist and facilitate wound healing.^26^ The percent wound area that remained, comparing CO-GEM to air respectively, 56.8% versus 68.6% at day 14; 25.1% versus 41.0% at day 28; and 11.8% versus 26.9% at day 42 (Figure 3D). By day 42, CO-GEM treatment accelerated wound healing by approximately 14 days (Figure 3D). Serous fluid from the wounds of the CO-GEM-treated and control pigs was removed on Day 12. The CO-GEM-treated wounds were found to have lower concentrations of pro-inflammatory cytokines (Figure S7). This acceleration of wound healing and reduction in pro-inflammatory cytokines were in line with our prior observations in diabetic mouse models.^13^ For direct product use, we envision the foams and hydrogels as being preserved and sold in pressurized individualized packages.

**Figure 4:**
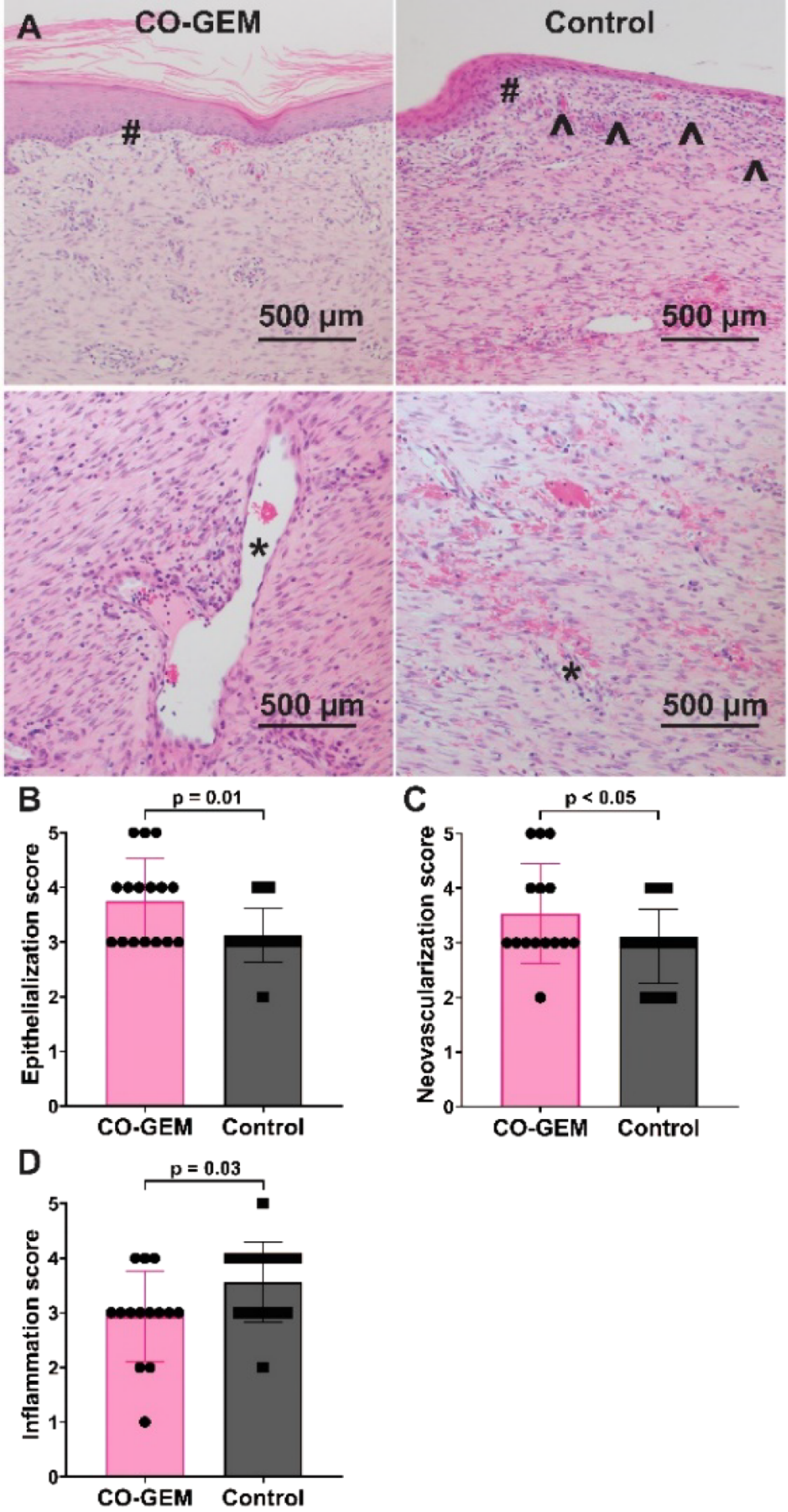
Histologic assessment of wounds at day 42 demonstrated improvement in epithelialization and neovascularization and reduction in inflammation. (A) Representative H&E histologic sections from CO-GEM-treated and control Tegaderm-treated animals. **#** indicates epithelialization with keratinization, **^** indicates epithelialization without keratinization and underlying scarring, and * indicates neovascularization. The neovascularization in the control group was less mature. (B) Scoring of epithelialization, neovascularization, and inflammation for tissue sections from all 8 wounds for each animal (*n* = 16 sections/arm). CO-GEM-treated wounds had significantly increased epithelialization and neovascularization scores and significantly reduced inflammation scores. *p* values were quantified by unpaired *t* test.

In addition, we performed histologic evaluation of skin biopsies by H&E, Masson’s trichrome, CD31 (endothelial marker), and α-smooth muscle actin (α-SMA) staining (Figure S8). Quantitation of CD31 staining demonstrated an increase in vascularization within the wound beds of skin in pigs treated with the CO-GEMs compared to Tegaderm (Figure S8A,B). In addition, there was a significant increase in α-SMA staining in the wound bed of skin in the CO-GEM-treated pigs compared to the Tegaderm-treated pigs (Figure S8A,C). Further, a blinded pathologist with expertise in wound healing, scored the H&E samples based upon epithelialization, neovascularization, inflammation, granulation tissue maturation, and collagen, using an established rubric.^27^ CO-GEM-treated skin was found to have increased epithelialization and neovascularization and a reduction in inflammation compared to control skin treated with Tegaderm alone (Figure 4A,B). There were nonsignificant increases in granulation tissue maturation and collagen (Figure S9A,B). Furthermore, the epithelialized portions of the skin were subject to mechanical testing (Figure S10A–D), and the skin treated with CO-GEMs was found to have nonsignificant increases in linear stiffness (Figure S10A) and max load (Figure S10B).

Given the wound healing benefit, clinical testing against other technologies will be necessary to demonstrate superior benefits over existing systems. The small sample size of the study limits the generalizability of the study and would benefit from repeating in a larger cohort of pigs, especially in comparison to other wound healing grafts shown in Table S1.^28−33^ In addition, testing in diabetic pigs will provide broader applicability in a disease setting where wound healing is significantly impaired.

### CO-GEMs Demonstrate a Favorable Safety Profile and Exhibit No Observable Toxicity

The translation of the CO-GEMs hinges entirely on the safety of the materials used for the topical delivery of CO. During the application of CO-GEMs, the pigs were weighed every 3–7 days and found to consistently gain weight throughout the course of the study (Figure S11A). Systemic levels of CO, as measured by carboxyhemoglobin (% COHb), were evaluated prior to, 15 min and 1 h after dosing the CO-GEMs. We found that COHb was well below the 14% limit set by the FDA for the inhaled CO trials (Figure S11B).^34^ It was noted that COHb was slightly elevated above baseline for the first 2–3 doses.

The materials tested here are recognized as GRAS by the FDA and also cost-effective.^35^ They are commonly used in general wound dressings and cosmetics.^36^ Additionally, all clinical trials with CO conducted to date have shown that CO treatment is very safe, particularly in immunocompromised patients, such as those with interstitial pulmonary fibrosis and ARDS.^34,37,38^ Despite this, emphasis will continue to be placed on safety measures to minimize toxicity since CO is present in the formulation.^21^

## Conclusions

Composite hyaluronic acid CO-GEMs prolong CO release and their application on full-thickness wounds in pigs resulted in accelerated wound healing. Further, CO-GEMs were found to be safe in large animal models with low systemic CO exposure. CO-GEMs have the potential to substantially accelerate healing in chronic wounds.

## Supporting Information Available

*In vitro* scratch assay, cell viability studies, additional photographs of GEM manufacturing techniques, material characterization of CO-GEMs, GC analysis of CO gas concentration in GEMs, demonstration of CO-GEM stability, cytokine/chemokine analysis of serous fluid at day 12, additional histologic evaluation of skin from porcine study, additional histologic scoring of wounds at day 42, mechanical testing of healed skin, and weights and COHb % in pigs (PDF)

## Abbreviations

GEM: gas-entrapping materials
CO: carbon monoxide
COHb: carboxyhemoglobin
NS: not significant
N: Newton
C: Celsius
ppm: parts per million.
