# Supplementary material for: Composite Hyaluronic Acid Gas-Entrapping Materials to Promote Wound Healing
Source: Biomacromolecules. 2025 Jan 2;26(1):201–8. doi: 10.1021/acs.biomac.4c00904 (PMC11733945; doi:10.1021/acs.biomac.4c00904)
Supplement: Supplementary file 1 — bm4c00904_si_001.pdf [file bm4c00904_si_001.pdf]

Supplemental Information

Composite Hyaluronic Acid Gas-Entrapping materials to Promote Wound Healing

Emily Witt[§,°], Emily B. Petersen[†], Eyas Alzayadneh[¶], Ryan J. Courtney[‡], Marc J. Brouillette[†], Qi Wang[§,‡], Maxwell Y. Sakyi[†], Nicole A. D. Watson[†], Dominic Rivas[†], Jianling Bi[§,°], Lindsey Culver[§], Kyle Balk[§,°], Colin Reis[§,°], Slyn Uaroon[€], Kaitlyn A. McClintic[§], Samual Hatfield[‡], Kristan S. Worthington[§], Edward A. Sander[§], Giovanni Traverso[#,&], Leo E. Otterbein[%], Jessica E. Goetz[†,°], Douglas C. Fredericks[†], James D. Byrne[§,°,]*

[§]Department of Biomedical Engineering, University of Iowa, Iowa City, Iowa, 52242, United States

[°]Department of Radiation Oncology, University of Iowa, Iowa City, Iowa, 52242, United States

[¶]Department of Pathology, University of Iowa, Iowa City, Iowa 52242, United States

[†]Department of Orthopedics and Rehabilitation, University of Iowa, Iowa City, Iowa, 52242, United States

[‡]Carver College of Medicine, University of Iowa, Iowa City, Iowa, 52242, United States

[€]Department of Otolaryngology, University of Iowa, Iowa City, Iowa, 52242, United States

[#]Division of Gastroenterology, Brigham and Women's Hospital, Harvard Medical School,

Boston, Massachusetts, 02115, United States

[&]Department of Mechanical Engineering, Massachusetts Institute of Technology, Cambridge, Massachusetts, 02139, United States

[%]Department of Surgery, Beth Israel Deaconess Medical Center, Harvard Medical School, Boston, Massachusetts, 02215, United States

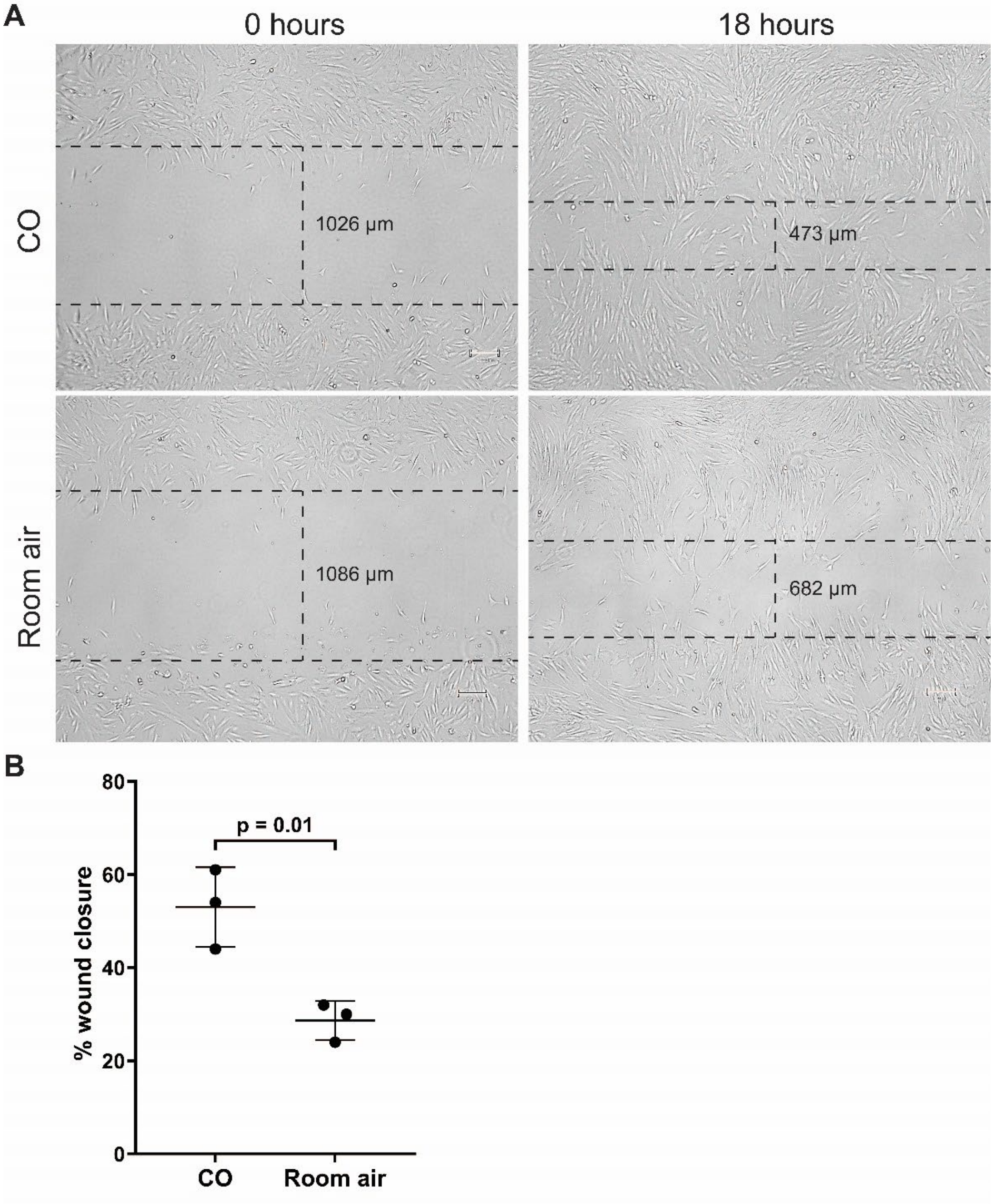

Figure S1. *In vitro* scratch assay using human dermal fibroblasts. (A) Microscopic images demonstrate increased wound closure at 18 hours under exposure of 250 ppm CO or normal incubator conditions. (B) Percent wound closure at 18 hours under exposure to 250 ppm CO compared to normal incubator conditions (n=3). P values were determined by unpaired T test.

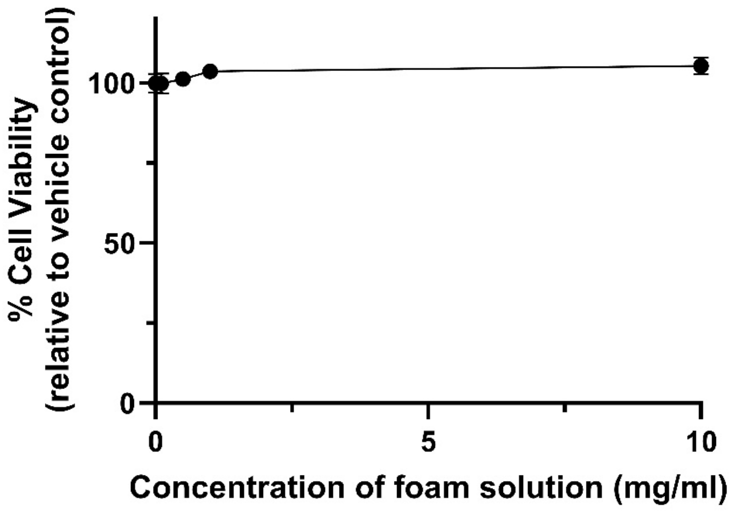

Figure S2. GEM pre-foam solution exhibited no cytotoxicity in human dermal fibroblasts up to 10 mg/mL (n = 8/concentration).

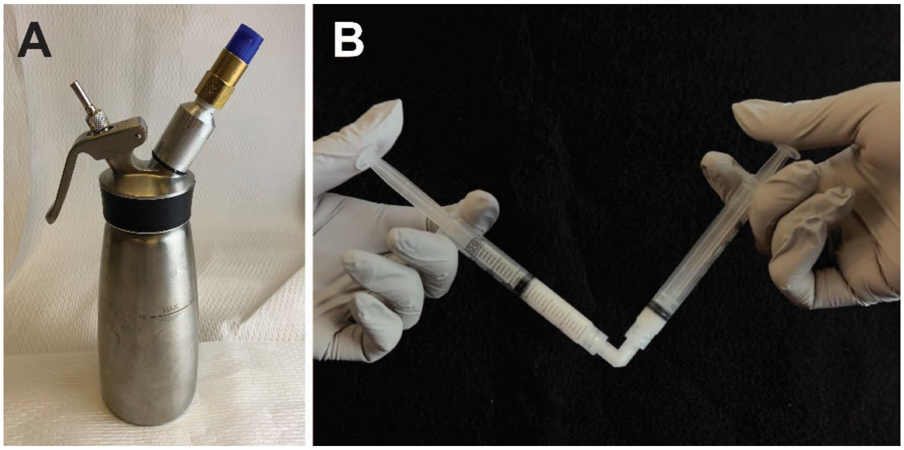

Figure S3. GEM manufacturing relies on simple methods traditionally used in cooking and small volume mixing. (A) Customized whipping siphon for generation of foam CO-GEMs. (B) Syringe mixing method for generation of CO-GEM hydrogels.

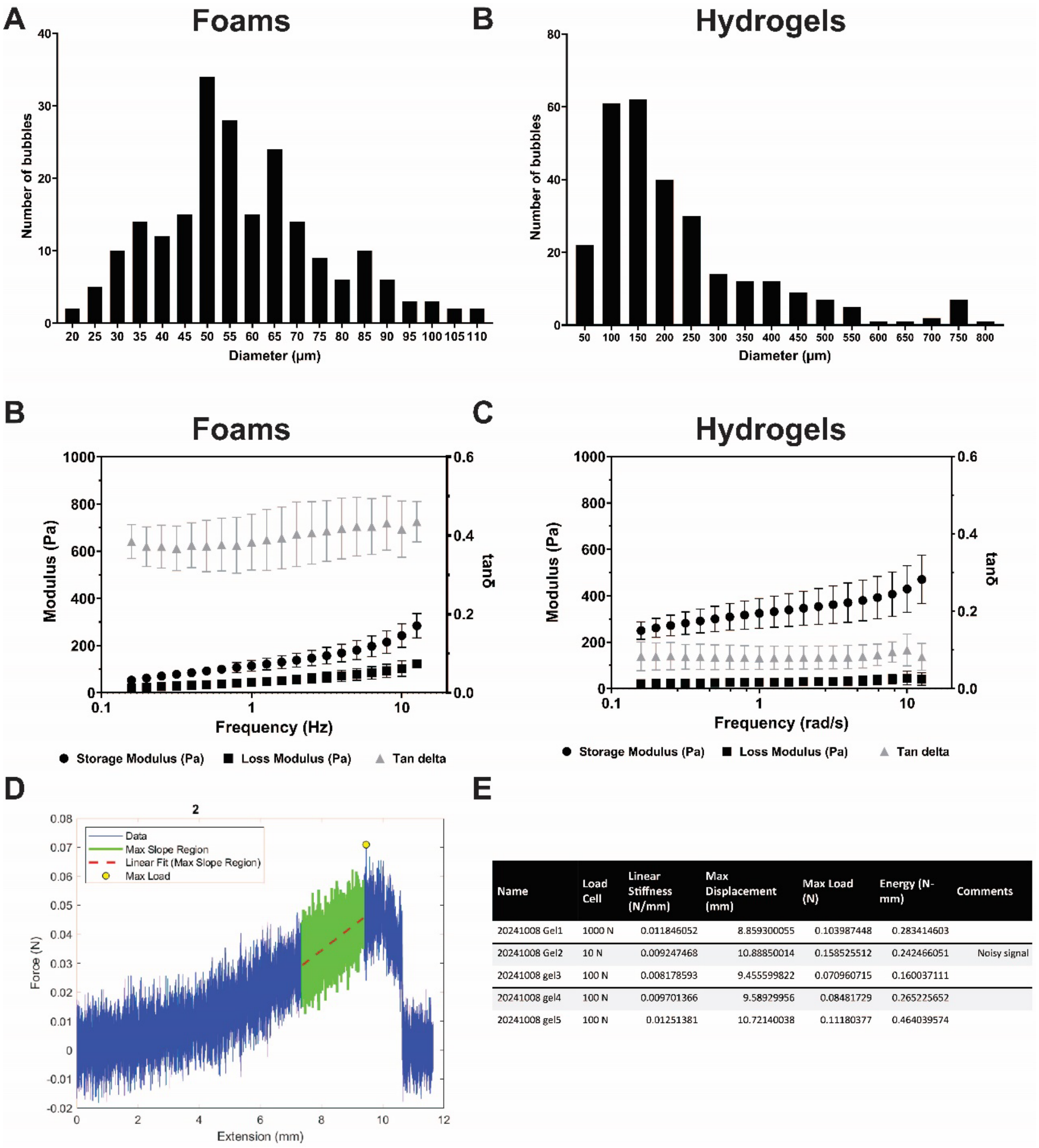

Name	Load Cell	Linear Stiffness (N/mm)	Max Displacement (mm)	Max Load (N)	Energy (N-mm)	Comments
20241008 Gel1	1000 N	0.011846052	8.859300055	0.103987448	0.283414603	
20241008 Gel2	10 N	0.009247468	10.88850014	0.158525512	0.242466051	Noisy signal
20241008 gel3	100 N	0.008178593	9.455599822	0.070960715	0.160037111	
20241008 gel4	100 N	0.009701366	9.58929956	0.08481729	0.265225652	
20241008 gel5	100 N	0.01251381	10.72140038	0.11180377	0.464039574	

Figure S4. Material characterization of CO-GEMs. (A) Histogram of CO bubble diameter in foam CO-GEMs. (B) Histogram of CO bubble diameter in hydrogel CO-GEMs. (C) Modulus of pressure as a function of strain (n = 3 per group) in foam CO-GEMs. (D) Modulus of pressure as a function of strain (n = 3 per group) in hydrogel CO-GEMs. (E) Representative force-displacement curve for hydrogel CO-GEMs. (F) Table of stiffness, displacement, and maximum load for hydrogel CO-GEMs (n = 5).

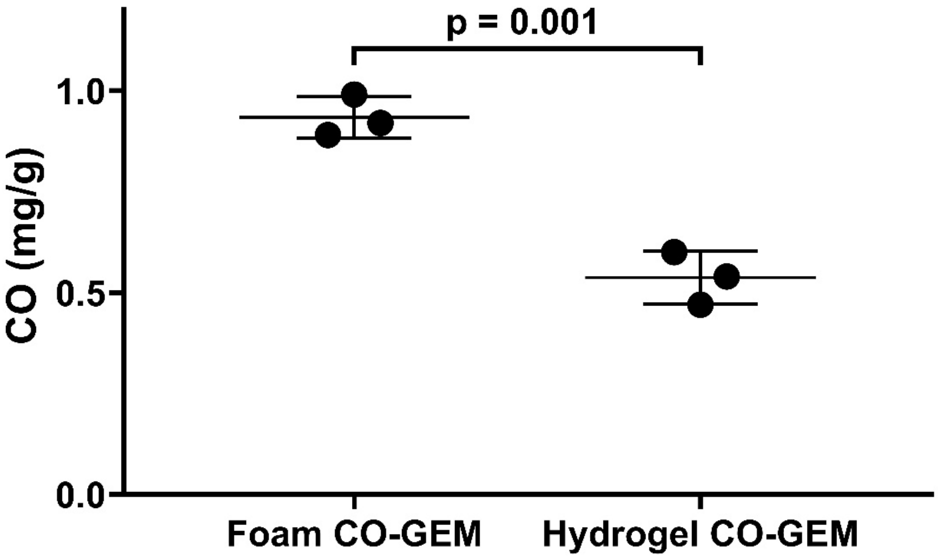

Figure S5. Foam CO-GEMs entrapped significantly higher CO amounts compared to hydrogel CO-GEMs. Results are mean ± standard deviation of n = 3 per group. P values were determined by unpaired t-test.

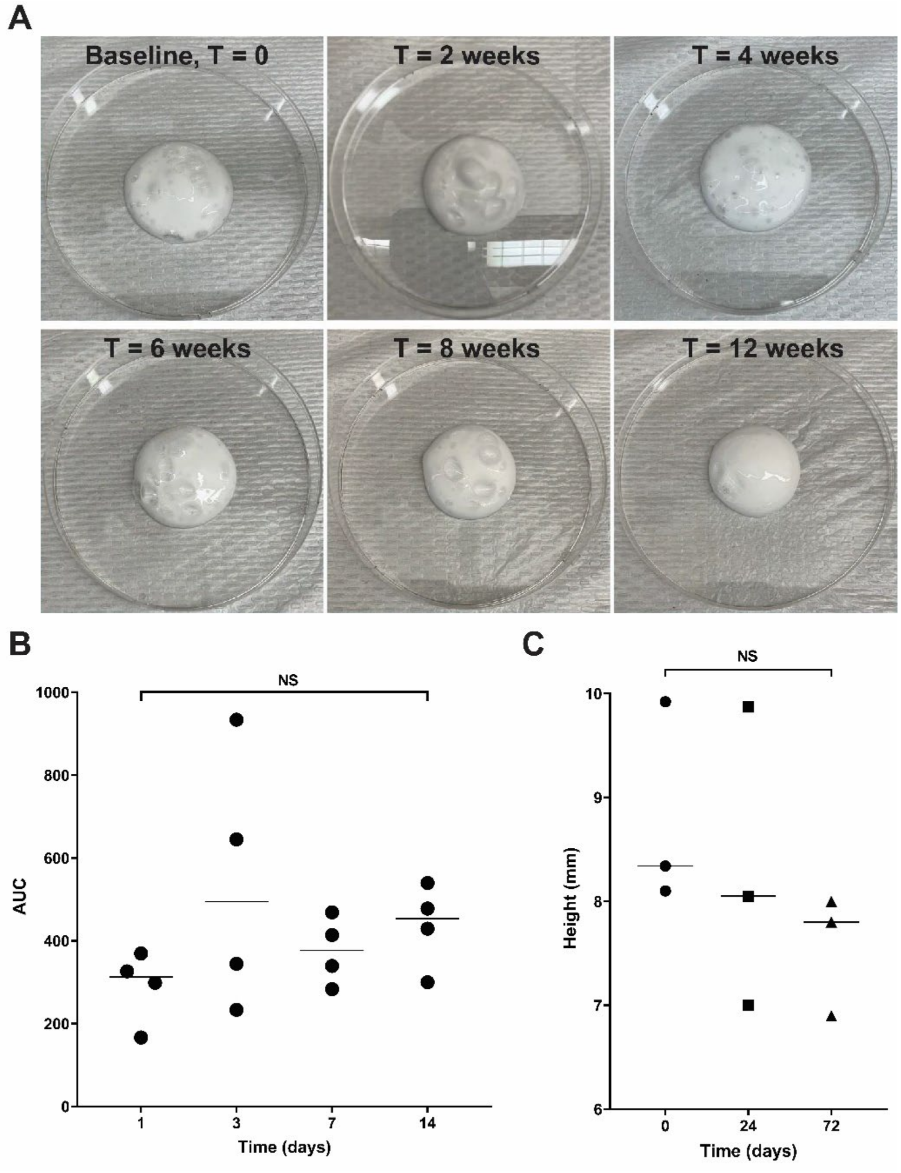

Figure S6. Demonstration of CO-GEM stability. (A) The whipping siphons with foam solution were maintained at 4 °Celsius over 12 weeks. The ability to generate reproducible foam GEMs were assessed and showed no change in foamability and macroscopic foam appearance. Reproduced with permission from AAAS.[18] (B) The hydrogels were maintained at -50 °Celsius over 2 weeks. The gas retention and delivery were assessed and showed no change in gas retention over 2 weeks. (C) Height of hydrogels maintained at 37 °Celsius in a humid chamber over 3 days.

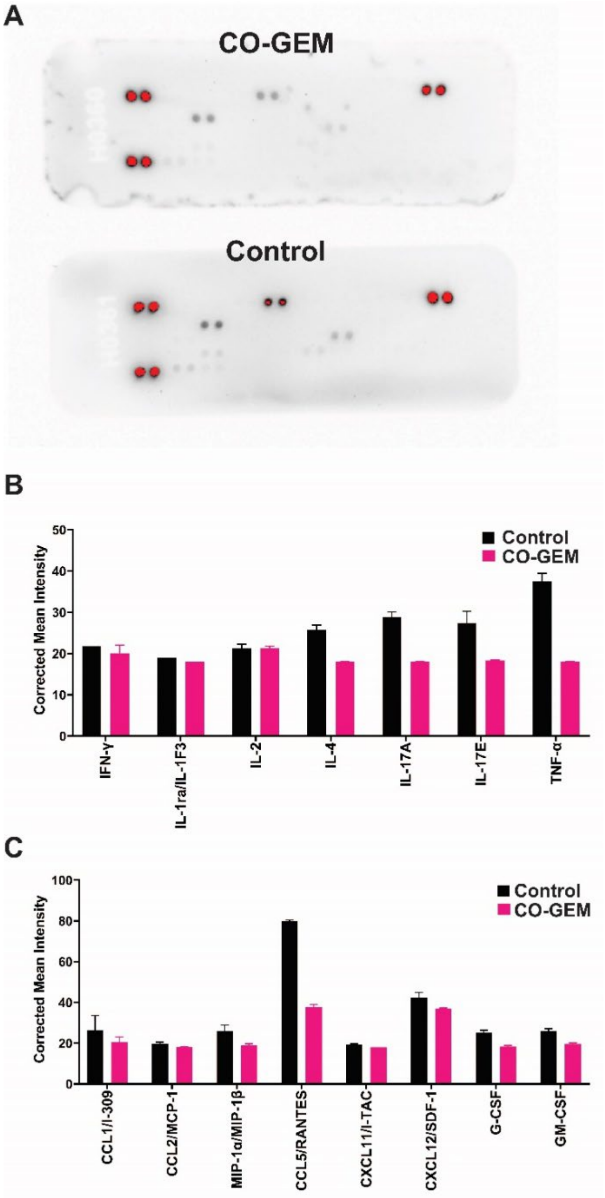

Figure S7. Effect of CO-GEM on cytokines expression in wound fluid. CO-GEM-treated wounds showed lower concentration of inflammatory cytokines and chemokines in serous fluid at day 12 compared to control wounds. (A) Cytokine/chemokine array from serous fluid from each wound was combined for each arm (control and CO-GEM; n = 2 runs/arm). Corrected mean intensity for (B) inflammatory cytokines and (C) chemokines in serous fluid. Statistics were not performed due to the qualitative nature of the analysis.

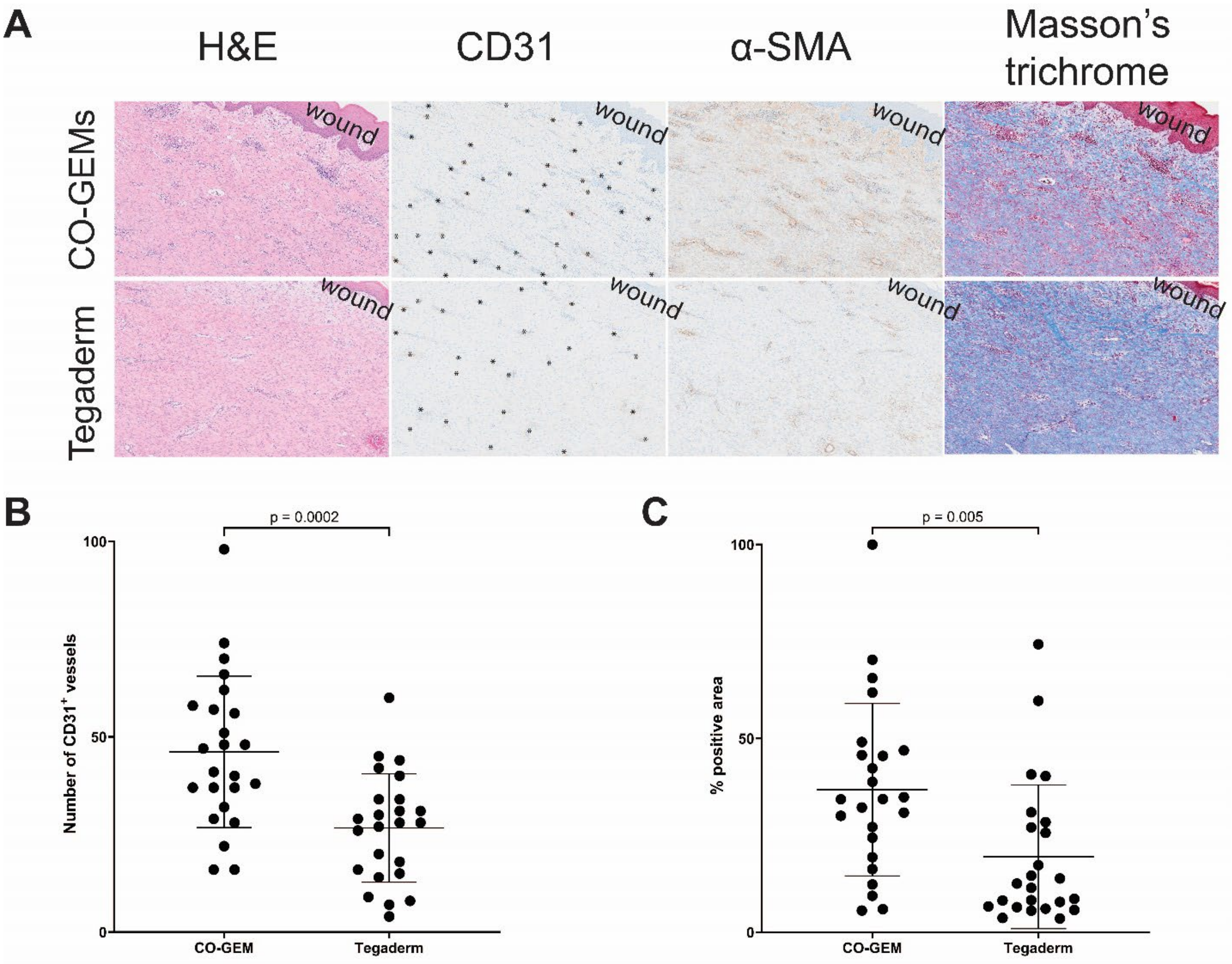

Figure S8. Histologic evaluation of healed porcine wound beds treated with CO-GEMs compared to Tegaderm. (A) Representative images of H&E, CD31, α-SMA, and Masson's trichrome stains. (B) Quantitation of CD31+ vessels per 4x field. (C) Quantitation of % positive area for α-SMA staining.

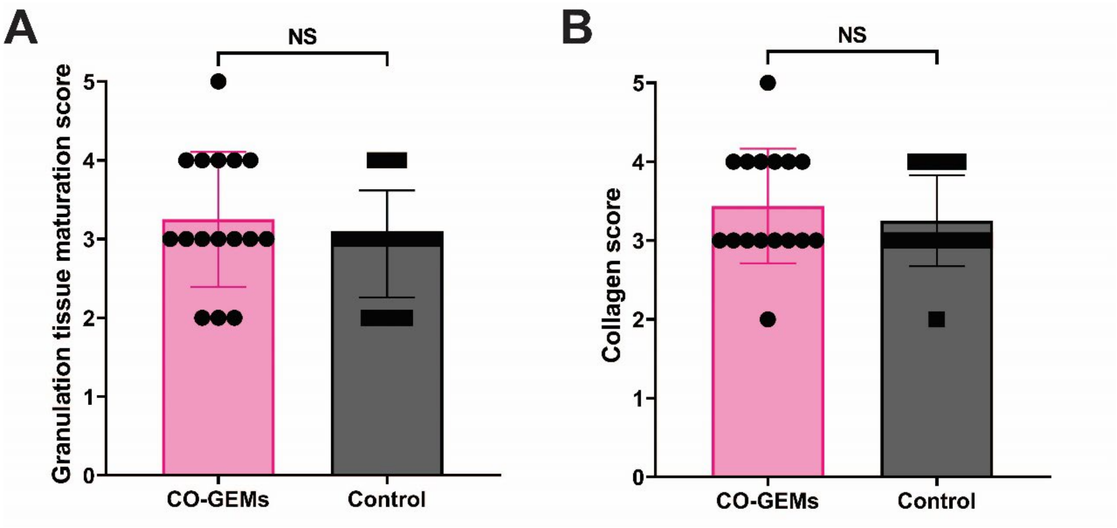

Figure S9. Histologic assessment of wounds at day 42 demonstrated non-significant improvement in (A) granulation tissue maturation and (B) collagen scores for tissue sections from all 8 wounds for each animal (n = 16 sections/treatment group). P values were determined by unpaired t-test. NS – not significant.

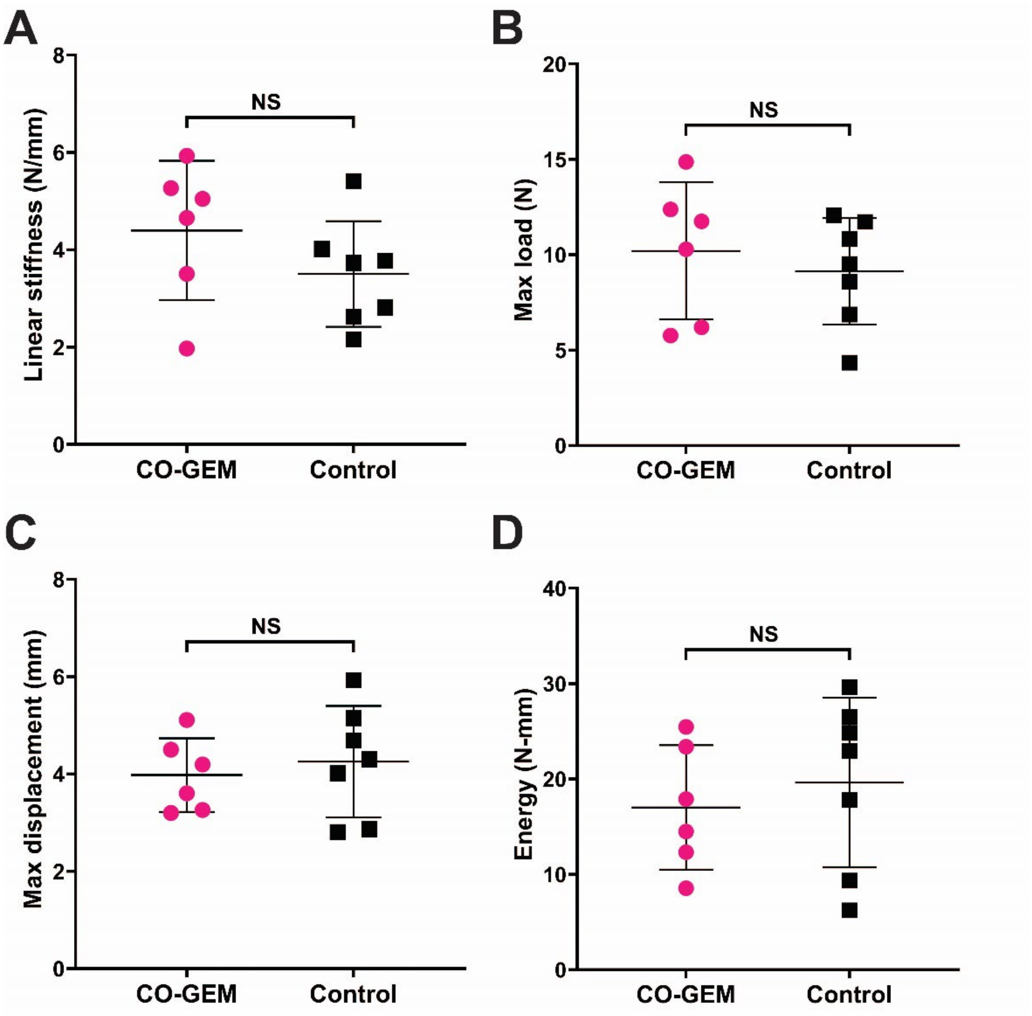

Figure S10. Effects of CO-GEM on tissue mechanics after healing. Mechanical testing of the healed skin tissue demonstrated that CO-GEM-treated wounds was stiffer and able to sustain a greater max load than control wounds. (A) Linear stiffness, (B) max load, (C) max displacement and (D) energy was determined for skin samples from control or CO-GEM-treated wounds at day 42 post-wounding (n = 6-7/treatment group). P values were determined by unpaired t-test. NS – not significant.

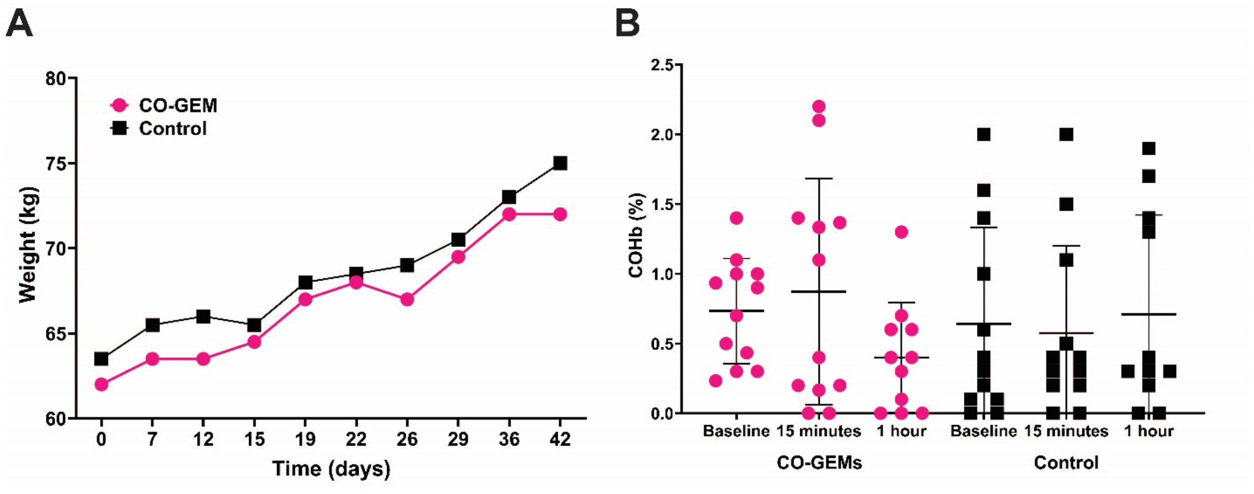

Figure S11. Effect of CO-GEM on pig body weight and COHb. (A) Pig weight over the course of study showed no significant difference in growth kinetics. (B) Carboxyhemoglobin (COHb%) over time measured before, 15 minutes post, and 1-hour post CO-GEM administration showed no elevation in systemic COHb compared to control.

Table S1. Clinically tested biomaterials used for wound healing.

Graft	Material composition	Indication	Demonstrated benefit in clinical trials	Reference
Integra BWM	Integra Dermal Regeneration Template (IDRT)	Diabetic wounds	Integra BWM reduced average wound healing time by 35 days. While also reducing the wound size 50% faster per week for the treatment group.	30
NovoSorb BTM	The combination of NovoSorb Biodegradable Temporizing Matrix (BTM) and RECELL Autologous Skin Cell Suspension (ASCS).	Burn	Once the NovoSorb BTM procedure was complete it took about two weeks for the skin to be healed. All trials showed acceptable healing and no wound infections.	32
Phoenix Wound Matrix	A 3D electro spun synthetic polymer matrix made up of nanofibers with pores about 5µm.	Burn, Acute, and Chronic Wounds	The Phoenix Wound Matrix healed 66% of wounds in 12 weeks. 38% of the wounds received only one application of treatment before being healed.	29
Restrata Wound Matrix	Synthetic matrix consisting of electro spun nonwoven nanofibers. The matrix is made up of polydioxanone and polygalactin 910.	Diabetic, post-surgery, and pressure ulcers	Restrata Wound Matrix is very biocompatible and reduces inflammation. By day 30 the wound size of the treatment group decreased by about 98% while the control group decreased by 64%.	33

Mirragen Advanced Wound Matrix	A flexible borate-based bioactive glass fiber wound matrix. This is a skin substitute made from natural body elements.	Chronic Wounds	The Mirragen Advanced Wound Matrix helped all patients achieve complete wound closure in an average of 55 days. It also lowered the average treatment cost by $84,186 and reduced wound duration by an average of 336 days.	28
SUPRA SDRM	A biodegradable polylactide-based matrix. It is synthetic and hydrolytically resorbable.	Chronic and difficult to heal wounds	The Supra SDRM showed an increase in cell migration to form new tissue and reduced hypertrophic scarring. Complete healing was seen within 12 weeks with weekly applications.	1
SUPRATHEL	An alloplastic synthetic microporous absorbable membrane that acts as a skin substitute.	Burns	The use of SUPRATHEL demonstrated high rates of spontaneous healing and reduced the need for surgical intervention. Wounds were completely healed in an average of 28 days.	31
NOP	An electrospun polymeric NO releasing patch with an ionic exchange resin	Diabetic wounds	No results posted	34
